# Social Workers in Pediatric Oncology: A Qualitative Study in Iranian Context

**DOI:** 10.31557/APJCP.2019.20.6.1871

**Published:** 2019

**Authors:** Leila Ostadhashemi, Maliheh Arshi, Maliheh Khalvati, Mostafa Eghlima, Hamid Reza Khankeh

**Affiliations:** 1 *Department of Social Work, *; 3 *Emergency and Disaster Health Department and Research Center in Emergency and Disaster Health, University of Social Welfare and Rehabilitation Sciences, *; 2 *Clinical Research Development Center of Panzdah-e Khordad Hospital, Shahid Beheshti University of Medical Sciences , *; 4 *Member of National Academy of Medical Sciences National coordinator, Tehran, Iran. *

**Keywords:** Pediatric oncology, cancer, social worker, burn out, stress

## Abstract

**Background:**

As professionals, social workers have a special position in relation to considering the needs of children with cancer and their families. Hence, it is important to recognize the experiences and challenges of social workers to improve care of their clients.

**Method:**

This study was a qualitative content analysis that aimed to determine a comprehensive understanding of pediatric oncology social workers’ experiences in Iran. In total, 19 social workers participated in the study. A purposeful sampling method was applied until reaching data saturation. Data were collected using semi-structured interviews and field observations. Then, the gathered data were analyzed through face content analysis. The study lasted from 2015 to 2017.

**Finding:**

Concepts extracted from social workers’ experiences consisted of the nature of oncology work, lack of professional competence, low organizational support and professional inferiority that were related to main concept of “exhausting and stressful service”. The results indicated that social workers’ involvement in stressful and emotionally demanding situations and facing with professional and organizational challenges caused personal exhaustion.

**Conclusion:**

In addition to explaining the social workers’ experiences and related factors, the results emphasize the importance of taking care of service providers to prevent them becoming stressed and exhausted. It is also important to protect patients from the consequences of stressed and exhausted care providers so further research is recommended to develop specific intervention.

## Introduction

Being diagnosed with and treated for cancer in children is potentially traumatic for children and their families. Family members and care givers experience a lot of financial, psychosocial and emotional problems and they need support, information, and advice (Rezaei et al., 2018; Yi et al., 2018). 

For many years oncology social workers have been considered the main providers of psychosocial services to cancer patients and their families around the world (Smith et al., 1998). As an effective member of a medical team, social workers participate in multidisciplinary teams and are among the first professionals to listen empathically to the needs and concerns of children and their families. They work for their clients as advocates for comprehensive care and for proper receipt of entitlements (Stearns et al., 2001; Jones, 2005). 

On the other hand, working in the field of cancer is a highly emotional experience for health care providers. Cancer patients are often in great need and deserving of important medical care as well as appropriate emotional and mental support (Saint-Louis, 2010). Professional caregivers are in contact with terminally ill patients and their families on a daily basis and they repeatedly experience death of the patients(Stearns et al., 2001; Rohan, 2009) . The end result of such long-term exposure to stress is often reflected in job burnout and fatigue (Saint-Louis, 2010; Yi et al., 2016).

It is well established that compassion fatigue and burn out among health providers and social workers affected their quality of life, clinical practices and quality of patient care. The psychological and physical symptoms of compassion fatigue and burn out reduce their sense of professional competence, capacity to bear others’ suffering, productivity and coping skills (Maslach, 2003; Yi et al., 2018). There are also economic reasons for considering, since work-related stress and fatigue is a major cause of job dissatisfaction, sickness absence and excessive staff turnover, which are costly to organizations and put professionals and patient’s health and safety at risk (van Wyk and Pillay Van Wyk, 2010; Sodeke-Gregson et al., 2013).

Significantly, to prevent these symptoms and consequences in pediatric oncology social workers, it is important to understand their experiences and challenges of their works. 

A review of the related literature in the field suggested limited knowledge and information relating experiences of social workers in cancer care (Simon et al., 2005). Previous studies, using various different approaches have addressed stress, job burnout and compassion fatigue among members of medical teams, mainly nurses, but only a few of studies have included social workers (Mukherjee et al., 2009; Najjar et al., 2009). There are relatively few studies on experiences of social workers in the field of cancer in general, as well as a shortage of research in pediatric oncology (Simon et al., 2005; Yi et al., 2016). And regarding the methodological nature of these studies (quantitative studies), experiences of pediatric oncology social workers (POSW) has yet to be evaluated. According to a review of the existing literature, no study can be found on experiences of POSW in Iran. To develop appropriate relevant prevention and care programs of POSW, it is essential to attain a description of their experiences in this field. 

*Objectives*

This study has been conducted in order to better understand POSW experiences in Iran and to highlight the challenges of nature of their work and service provision for children with cancer and their families.

## Materials and Methods

*Study design*

This study was a qualitative research using the 5-stage content analysis method as recommended by Grancheim and Lundmen (Graneheim and Lundman, 2004). Data were collected with no pre-assumptions and focused on participants’ responses. In the analysis, codes and concepts were extracted from social workers’ experiences using the inductive approach.

*Setting and Participants*

19 social workers were participated in the study, based on the three inclusion criteria: participants had to have degree in social work, five years’ work experience in pediatric oncology and a willingness to participate in the research (Table 1). Eligible participants were recruited through the lists of the Ministry of Health and Medical Education’s social workers. The first author contacted the participants and after declaring their willingness, she set the time of the interviews. Interviews were conducted with social workers with first hand and rich experience and who were articulate in expressing their experiences. All interviews were conducted with social workers working in Tehran hospitals at their workplace and 4 interviews were conducted with social workers from other provinces who attended the annual MAHAK conference held in Tehran in November 2015.In this study, sampling started purposeful and continued by theoretical until saturation was reached.

*Data collection*

Data were collected using in-depth semi-structured interview and field observation. A few open-ended questions were used to guide interviews by the research team. These focused on social workers’ experiences and the challenges they faced working in pediatric oncology. The interview questions evolved during the process of the study. The following general questions were asked to identify participants’ experiences:

1. Please talk about your experience of working in pediatric oncology.

2. Which challenges do you face in providing services to children with cancer and their families? 

In addition, complementary questions were added when necessary for example: “*Could you elaborate more on your experience?*” Participants were interviewed once, but 3 of the social workers, due to creating new questions, not answering some questions and in the case of an incomplete interview, the interview process were performed twice. In total, 22 interviews were conducted with participants. Two eligible social workers declined interviews for reasons of having a high work volume and unwillingness to be interviewed. On average, interviews lasted for about 40 to 60 minutes. All interviews were conducted by the first author, recorded (with consent of participants), transcribed and analyzed according to the principles of the content analysis method. The interviewer had sufficient expertise in conducting qualitative research and had received training on related qualitative educational courses and workshops.

*Data Analysis*

Data analysis was carried out according to the content analysis method as recommended by Grancheim and Lundmen (Graneheim and Lundman, 2004). Concepts were developed using the constant comparative analysis method. Data were coded and analyzed from the start of the data collection process. Coding stages were performed with an emphasis on constant data comparison, asking more detailed questions and writing memos during the interviews. Firstly, each recorded interview was transcribed in a text file, and a line-by-line review was done to extract data meaning units and codes. Then, concepts were formed by comparison of codes to determine an understanding of their common features and differences. Then, the researcher continued writing memos and asking deeper questions until all properties and dimensions had been determined and the researcher was convinced that saturation had been achieved. 

*Trustworthiness*

In this study, study criteria were determined as creditability, transferability, dependability and conformability (Polit and Beck, 2003). The strategy for gaining trust was to allow enough time to collect and analyze data, and to use multiple methods to collect information; including interviews and field observations (triangulation). Data and results of analyses were checked by some participants (member checking). Reviews were done by second, third and last authors and two doctoral students of social work with sufficient experience of conducting qualitative research.

*Ethical Considerations*

This research project was approved by the Ethics Committee of the University of Social Welfare and Rehabilitation Sciences (code: USWR.REC.1393.185) in January 2015. At the beginning of the interview, the purpose of the study, and data confidentiality were explained to the subjects and written consent was given. The study participants had the right to withdraw from the study at any stage.

## Results

**Figure 1:**
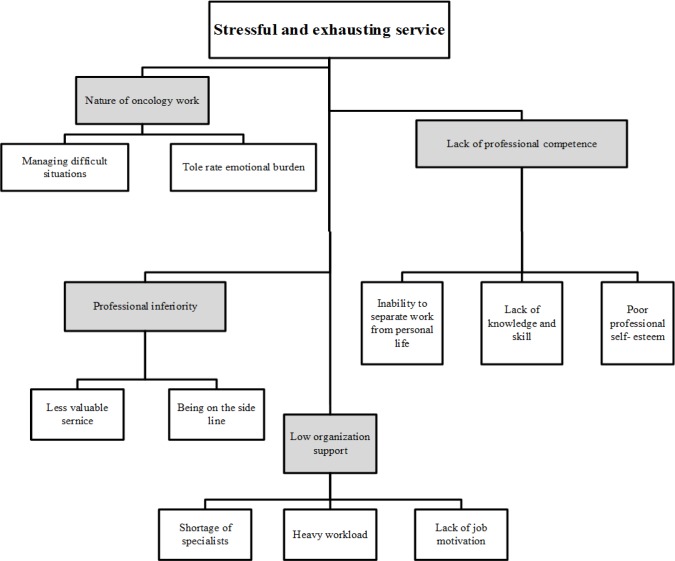
Four Key Concepts of Stressful and Exhausting Service

Four concepts were extracted from Data analysis, that described social workers’ experiences;*”* *nature of oncology work”, “professional inferiority”, “lack of professional competence*” and “*low organizational support*”; and their main concern was determined as working in a *“stressful and exhausting service”*) (Figure 1).

*First Theme: Nature of oncology work*

Social workers serving children with cancer and their families experience many difficulties and suffer great sorrow so common activities were described as *“Manage difficult situations”* and *“tolerate emotional burden”*.

*Managing difficult situations*

This concept presented an indication of social workers’ unpleasant experiences in managing critical situations, and being with children and families during difficult moments including suffering and despair. Social workers mentioned *“expressing bad news”*, *“working with traumatized families”* and *“being with children and families at the end of life “ *in relation to this concept. Although other members of a medical team are responsible for relating news of a diagnosis of child illness, they have very limited time so it is often the social workers that are left to support families in such circumstances. A social worker said about announcing unpleasant news:

“*Sometimes the family does not know or accept the child illness, and come to you to say it is not true. But you should say yes. Or when they hope cancer will be improved but it relapses. It is really hard to say for me*”.

A participant said the following in relation to working with a traumatized family and managing critical conditions:

“*When the child died, here was my shift. It was midnight, the mother was so upset, I calmed her down but suddenly found she had taken20 pills. I should handle it alone”*.

In relation to social workers’ experiences of being with a dying child and his or her family and involvement in their suffering in moments of despair, one social worker said:

“*When the family fined there is no hope, they tolerate great suffering and are not calm. We are always close to them. Sometimes the child says how he concerns his mother and wants to know what will happen. The mother is upset at that time, cries and is not calm*”. 

*Tolerate the emotional burde*

pediatric oncology social workers work with children below 15 years old and their families. They feel heavily burdened “*affected by children’s pain*” and “*involved in family emotions*”. Participants regarding kindness to children; described their pain and sorrow as annoying. In the case of being affected by a child’s pain, a social worker said:

“*Children are lovely and their pain affects you more. They tolerate a lot of pain in the whole treatment, seeing their pain is really annoying. I cannot tolerate it*”.

Social workers, by continuous assistance with the family during the care process, establish close and long-standing relationships and become involved emotionally with such families; this was expressed by a participant as follows:

“*You know you are always with them, you laugh with their laugh and you cry with their cry. You are next to a mother whose child died and she is singing lullabies, you try to calm her down but you yourself cannot tolerate*”.

*Second Theme: Professional inferiority*

According to participants’ experiences, social workers’ services are considered as less valuable than other medical services, both from the perspective of their clients and by other members of a medical team. *“Social workers on the sideline” *and *“less valuable service”* were deduced in our analysis of professional inferiority.

*Being on the sideline*

This concept indicates that physicians had key role in treatment decisions and social workers were excluded by the medical teams. Analysis determined it was concluded from the concepts *“physician-dominated care structure”* and *“excluded by the medical team”*. Social workers considered that treating children with cancer was one-dimensional and incomprehensive. A study participant mentioned the following in relation to the dominance of physicians in decision-making and treatment planning:

*“We just have a medical protocol in treatment plan but not for social work services. A doctor formulates a protocol, prescribes medicines and does the treatment. A patient’s psychosocial problems are not seen at all”*.

Another participant said about unwillingness among members of medical teams to foster interest in doing teamwork expressed by social workers:

*“Team work is in a way that a social worker tries to be close to the team to participate in the treatment process. But often physicians disagree with a social worker’s presence in the ward. Nurses guard against us and do not take us seriously”*.

*Less valuable service*

This concept has been concluded from the codes *“easy and free access to the service”* of social workers and their *“reductionist understanding of services”* from their clients’ point of view. Social workers were considered as always available and ready to provide a service at no cost and without demands; a participant mentioned that this contributed to a perception of social work as insignificant:

*“Always being available is against being professional. Given that a social worker should not ask for any cost and tolerate, this makes the service worthless. We are always with the family that sometimes the family draw back”*.

A social worker said the following about the perception of a reductionist understanding of their services from clients’ point of view: 

*“The appearance of our service is mostly identified by financial problems. The family understands that a social worker is a person to who they should refer to for financial problems. He/ she is the one to whom they refer when they have problems with treatment costs, travel costs, etc.”*.

*Third Theme*

*Lack of professional competence*

Complexity and diversity of the psychosocial problems related to childhood cancer is the field of the demand formation of specialists, experienced and skilled social workers to provide more effective assessments and interventions in the care process. *“lack of knowledge and skill”*, *“poor professional self-esteem”* and *“inability to separate work from personal life”* were deduced in our analysis of this concept.

*Lack of knowledge and skill*

This concept indicates inadequate knowledge and specialized skills of social workers to provide a high quality and appropriate service to children with cancer and their families. It includes the concepts *“lack of related knowledge”* and *“poor intervention skills”*. Regarding lack of knowledge and skills, participants considered poor specialized training at university, inadequate retraining and related resources and lack of specialist professors. For example:

*“Our field of study has no professional orientation. Our social worker’s information about childhood cancer is little, they have no university training. We have no related Persian references and professors for education”*.

Social workers have highlighted their shortage of skills for implementation of specialized interventions, particularly interventions of grief and end of life care and mentioned that have unable to offer the appropriate support to families; this was expressed by a social worker as follows: 

*“The day Ali died, I cried the distance between my work place and home. The imagination of his mother coming to me made me mad. When she came, I could say and do nothing just crying, she cried too”*.

*Poor professional self-esteem*

This concept indicates a* “lack of confidence in the ability”* and *“inability to introduce the profession” *by social workers. Participants mentioned uncertainty in relation to their professional ability and capacity; one of the social workers mentioned a lack professional competence as follows: 

*“We ourselves do not believe in our abilities and our low self-esteem and confidence when working causes referring families to other specialists and we do not interfere”*.

Social workers expressed that they have not succeeded in introducing the profession and making contributions to the team; this was expressed as follows:

*“Unfortunately we cannot present ourselves. We did not show that we work with the family and social structure. If a psychologist intervenes in the field of psychology, a physician intervenes in the field of the body, my expertise is in the field of the society”*.

*Inability to separate work from personal life*

Two concepts of *“carrying burden of sorrow” and “transmission of negative emotions to the family” *indicated social workers’ in ability to manage the effects of unpleasant experiences of their work place on other aspects of their lives. A social worker said the following in relation to carrying the burden of sorrow:

*“I desire when going out put everything behind but it is not possible. Sometimes I sit and cry for children without informing others”*.

Another participant said about her experience of conveying negative emotions to the family:

*“The day Amir Hossein died, I was so upset that at home I got mad at everyone”*.

*Fourth Theme*

*Low organizational support*

Social workers have described a low level of institutional support from their work place and complained of high workloads, delegating non-specialized tasks as well as a lack of attention from managers to their work pressures and low salaries. This concept included *“shortage of specialists”*, *“heavy workload” *and *“lack of job motivation”*.

*Shortage of specialists*

This concept indicates *“hiring non-specialists”* and *“lack of experienced staff workforces”* in pediatric oncology. Appointing graduates from other disciplines to positions of social work in medical centers was mentioned by one of the social workers as follows:

*“In many but not all hospitals, some have no social work degree. In towns, one in archive department now works as a social worker, so how can she do it?”*

Inexperienced social workers have increased the workload of the more experienced workers. In this regard, one of them said:

*“Social workers here are often newcomers and take great energy. You should do your work and teach them”*.

*Heavy workload*

Social workers in some medical centers were obliged to provide a service to all medical wards so they had many patients. And they were often forced to do unrelated tasks rather than engage in specialized activities. *“The number of patients assigned” *and *“delegating non-specialized tasks“ *were identified as concepts that implied a heavy workload. For example, a social worker said the following about the number of patients assigned:

*“Here, I regard all wards because I am the only social worker for all wards. The number of social workers is small compared to patients. I do not reduce my work so there is more pressure on me”*.

Also, another social worker said the following about his experience of non-specialized tasks:

*“Here, a social worker prepares medicines, all of us neither passed a course of pharmacology nor know medicines. But here we should recognize medicines and know the price and manufacturer”*.

*Lack of job motivation*

This concept indicated two concepts of *“inadequate salary”* of social workers and *“ignoring psychological needs and work pressure”* by institutions. One social worker considered economic problems as having the biggest effect on the service mentioned the problem of a low salary; her words are quoted below:

*“Here, we have the least salary even with the work difficulty. The concern when the life economic problems are not resolved naturally disturbs peace of mind and affects the work”*.

Also another participant said the following about the organization’s inattention to psychological needs and work pressure:

*“Here, the work in addition to the body involves the mind. I should not say, but here you have no right to talk about the work pressure and needs, even they do not think of you”*.

**Table 1:** Demographic Characteristics of Participants

Gender	Position	Age	Job experience
Female	Supervisor	45-36	20
	Supervisor	45-36	10
	Supervisor	35-26	9
	Supervisor	35-26	5
	Social worker	35-26	7
	Social worker	35-26	6
	Social worker	35-26	6
	Social worker	35-26	9
	Social worker	35-26	5
	Supervisor	55-46	25
	Social worker	35-26	8
	Supervisor	55-46	30
	Supervisor	45-36	9
	Supervisor	45-36	14
	Social worker	45-36	9
Male	Social worker	35-26	5
	Social worker	45-36	8
	Supervisor	35-26	8
	Social worker	35-26	12

## Discussion

The present study applied qualitative content analysis to gain a deep understanding of experiences of POSW. This study was also the first qualitative study to describe experiences of the social workers that provide services in the field of pediatric oncology in Iranian hospitals. The concepts extracted from this research included the nature of oncology work, professional inferiority, lack of professional competence and low organizational support, related to the concept of *“exhausting and stressful service”*.

The results of this study suggested that social workers in pediatric oncology experience relatively high levels of work-related stress and burn out. Likewise, previous studies on the work of social workers in the field of cancer have identified compassion fatigue, vicarious traumatization, secondary traumatic stress and burnout (Simon et al., 2005; Yi et al., 2016). 

The findings of this study were in accordance with results of similar studies in that the nature of oncology work was described as stressful and exhausting because it involves unpleasant events associated with cancer treatment, talking about adverse events, working with terminally ill patients and supporting clients who have experienced trauma (Dean, 1998; Alkema et al., 2008; Whitebird et al., 2013).

Other causes of stress reported by social workers were the emotional burden of a child’s pain during intensive-associated treatment and involvement in a family’s emotions as POSW developed long-term relationships with families of patients in oncology. Yi et al., (2016) found that social workers in oncology were heavily burdened by difficult tasks such as talking about death, assisting clients to make end-of-life plans, or comforting families after death of a loved one. Other studies also reported that establishing long-term and close relationships and empathic engagement with a cancer patient and his or her family had a heavy emotional burden and induced compassion fatigue for the service providers (Cho and Jung, 2014; Lee and Min, 2014). 

Professional inferiority was another concept that was identified from social workers’ experiences. POSW indicated the key role of physicians in formulating treatment protocol, ignoring clients’ psychosocial needs in care programs and POSW barely participated in formulation and implementation of care plans. Borland (1981); Kadushin and Kulys (1995) and Silva et al., (2015) indicated weakness among social workers to control the physician-dominated team structure . 

Social workers were not readily accepted as members of a medical team and they faced a one-sided effort for integration. Kadushin and Kulys (1995) and Reid et al., (1999) reported that members of multidisciplinary teams did not understand the role of social workers and did not appreciate what they could accomplish.

According to this study POSW were always available for clients and they have considered this problematic because on the one hand, their work is effective on supporting clients and on the other, it is considered as the cause of their undervalued professional services. Social worker services have also been regarded by clients in reductionist way and expressed understanding and non-specialized opinions of their work. Reid et al., (1999) showed that social workers reported that their role in mental health was misunderstood and not adequately valued by others. 

A lack of professional competence in social workers included concepts of a lack of knowledge and skill, poor professional self-esteem and an inability to separate work from life all contributed to difficulty in managing cases and work situations; and caused high levels of stress and exhaustion. The results of this study indicate a lack of related training and education available for social workers, especially for end of life care and managing family grief. The results of Yi et al., (2016) also confirmed poor knowledge and skill among social workers in oncology in Korea. Jones (2005) and Zebrack et al., (2008) have indicated a lack of specialized knowledge among social workers for end of life care. However, poor knowledge and skill among social workers and their inability to provide the specialized services have contributed to a lack of professional self-esteem. Another influential factor affecting this lack of professional competence is an inability to separate work from home life. The results of this study, in line with other reports show that the effect of unpleasant work experienced by social workers and their inability to manage these experiences had an effect on their personal life and caused burnout (Simon et al., 2005; Yi et al., 2016).

According to participants’ reports, they received limited organizational support from their institutions. Hiring social workers with no related academic training, a lack of specialized and experienced workforce, delegating unrelated and non-specialized tasks out of professional duties of social workers and many patients burdened social workers with high workloads. In addition to fatigue and exhaustion, they spent a lot of time doing non-professional activities and failed to carry out their professional duties and provide a high-quality service in accordance with the needs of their clients. Yi et al., (2016) indicated that delegating non-related tasks and a heavy workload caused fatigue among social workers in oncology. Davis et al., (2008) mentioned a lack of personnel and time limitations as obstacles of pediatric care. Findings considered ignoring financial and psychological needs of service providers and work pressures as factors causing burn out among professional caregivers. Several studies have considered the above effective on burnout (Rabin and Zelner, 1992; Cushman et al., 1995; Gelinas et al., 2012).

In conclusions this study shows that various factors affected social workers’ experiences in providing services for children with cancer and their families. Results indicate that their experiences, due to continuous involvement in shock, pain, sorrow and grief of children and their families, were stressful and exhausting. The mentioned concerns, together with minimum organizational support, lack of job value and inadequate knowledge and skills, lead to job ‘burnout’, which seems to be a serious matter for the health of POSW.

Social workers are recognized as crucial members of multidisciplinary teams in oncology treatment centers due to the type of service they provide. Thus, managers and administrators of health systems should consider the impact of pressure and tension of their work on physical and psychological health of these service providers, and implement policies that take care of them and improve the quality of social services. Also, given the lack of professional competence of POSW associated with semiprofessional role and the high level of stress that is imposed on them, it is recommended that more education and training be provided for social workers in order to reduce work-related stress and exhaustion.

Given that the present study was the first on pediatric oncology in Iran, it is important to analyze these results with caution and to be aware of limitations. This study focused on the challenges of providing service clients, but POSW might also experience satisfaction and growth. Future studies could explore ways of coping with work-related stress and burn out in POSW. we did not consider how participants’ demographic characteristics were related to their experiences. Although the results, considering the use of qualitative approach in the study, cannot be generalized to relate all countries, they may be applied to analogous cultures and situations. It is suggested that future research explores the experiences of POSW in other cultures.

## Ethical issues (Consent for publication)

Participants agree to publish data without mentioning their names and personal information. They also became aware of the research results.

## Funding

The authors received no financial support for the research, authorship, and publication of this article.

## Authors’ contributions

All authors contributed to writing, editing and revision of the manuscript and approved the final manuscript. First, second, third and correspond authors led the design of the data collection methods and analysis.

## Competing interests

The authors declared no potential conflicts of interest with respect to the research, authorship, and publication of this article.

## References

[B1] Alkema K, Linton JM, Davies R (2008). A study of the relationship between self-care, compassion satisfaction, compassion fatigue, and burnout among hospice professionals. J Soc Work End Life Palliat Care.

[B2] Cho HJ, Jung MS (2014). Effect of empathy, resilience, self-care on compassion fatigue in oncology nurses. J Korean Acad Nurs Adm.

[B3] Cushman L, Evans P, Namerow P (1995). Occupational stress among aids social service providers. Soc Work Health Care.

[B4] Dean RA (1998). Occupational stress in hospice care: Causes and coping strategies. Am J Hosp Palliat Care.

[B5] Gelinas C, Fillion L, Robitaille MA (2012). Stressors experienced by nurses providing end-of-life palliative care in the intensive care unit. Can J Nurs Res.

[B6] Graneheim UH, Lundman B (2004). Qualitative content analysis in nursing research: concepts, procedures and measures to achieve trustworthiness. Nurse Educ Today24.

[B7] Jones BL (2005). Pediatric palliative and end of life care: The role of social work in pediatric oncology. J Soc Work End Life Palliat Care.

[B8] Lee HJ, Min HS (2014). The influential factors on compassion fatigue in hospital nurses. J Muscle Joint Health.

[B9] Maslach C (2003). Job burnout: New directions in research and intervention. Curr Dir Psychol Sci.

[B10] Mukherjee S, Beresford B, Glaser A (2009). Burnout, psychiatric morbidity, and work-related sources of stress in pediatric oncology staff: a review of the literature. Psychooncology.

[B11] Najjar N, Davis LW, Beck-Coon K (2009). Compassion fatigue: A review of the research to date and relevance to cancer-care providers. J Health Psychol.

[B13] Rabin C, Zelner D (1992). The role of assertiveness in clarifying roles and strengthening job satisfaction of social workers in multidisciplinary mental health settings. Br J Soc Work.

[B14] Rezaei Z, Sani M, Ostadhashemi L (2018). Quality of life of mothers of children with cancer in Iran. Koomesh.

[B16] Simon C, Pryce J, Roff L (2005). Secondary traumatic stress and oncology social work: Protecting compassion from fatigue and compromising the worker’s worldview. J Psychosoc Oncol.

[B18] Sodeke-Gregson EA, Holttum S, Billings J (2013). Compassion satisfaction, burnout, and secondary traumatic stress in UK therapists who work with adult trauma clients. Eur J Psychotraumatol.

[B20] van Wyk BE, Pillay Van Wyk V (2010). Preventive staff support interventions for health workers. Cochrane Database of Systematic Reviews.

[B21] Whitebird RR, Asche SE, Thompson GL (2013). Stress, burnout, compassion fatigue, and mental health in hospice workers in Minnesota. J Palliat Med.

[B22] Yi J, Kim J, Akter J (2018). Pediatric oncology social workers’ experience of compassion fatigue. J Psychosoc Oncol.

[B23] Yi J, Kim MA, Choi K (2016). When does compassion fatigue hit social workers? Caring for oncology patients in Korea. Qualitative Social Work.

